# How to improve informed consent processes in clinical trials with cancer patients: a qualitative analysis of multidisciplinary experts’ perspectives

**DOI:** 10.1186/s12910-025-01348-5

**Published:** 2025-12-02

**Authors:** Christine Bernardi, Daniel Wolff, Florian Lüke, Johannes Hies, Ruth Horn, Frederike Seitz, Daniel Heudobler, Wolfgang Herr, Anne Herrmann

**Affiliations:** 1 Medical Sociology, Department for Epidemiology and Preventive Medicine, University of Regensburg, Regensburg, Germany; 2 Department of Internal Medicine III, University Hospital Regensburg, Regensburg, Germany; 3 Legal Department, University Hospital Regensburg, Regensburg, Germany; 4 Institute for Ethics and History of Health in Society, Medical Faculty, University of Augsburg, Augsburg, Germany; 5 Ethox Centre, Nuffield of Population Health, University of Oxford, Oxford, UK; 6 Ethics Committee, University of Regensburg, Regensburg, Germany; 7 Bavarian Cancer Research Center, Regensburg, Germany

**Keywords:** Oncology, Clinical trial, Informed consent, Ethics, Semi-structured interviews

## Abstract

**Objective:**

According to legal and ethical obligations, patients must be thoroughly informed about the trial in which they could enrol, requiring them to consider and digest a significant amount of complex information. Many cancer patients feel overwhelmed which hinders their ability to make informed decision regarding their care. There is a need for further evidence-based strategies on how to improve both physician-patient-communication and informed consent (IC) documents in this area. We explored the views of experts from various disciplines on communication in IC processes in oncology.

**Methods:**

Seventeen semi-structured interviews with multidisciplinary experts were conducted and analysed using framework analysis.

**Results:**

Several experts stated that IC documents have become highly legalistic, often prioritizing the interests of sponsors and further institutions involved over patient understanding. IC conversations are considered essential, as many patients do not thoroughly read IC documents. Conducting an unbiased IC conversation in an understandable manner may be challenging for physicians because they often have vested interests in recruiting patients for trials. Introducing evidence-based checklists for IC conversations and involving nursing staff may assist in addressing practical issues patients may have, reduce anxiety, and increase consent rates. Strategies to improve IC documents include reducing potentially irrelevant information (e.g., on contraception), improving the adaptation of international consent forms to local settings and incorporating graphical abstracts and study flowcharts to offer brief and visually engaging summaries. Additionally, fostering open dialogue and involvement of all relevant stakeholders (including patient representatives from various sociodemographic backgrounds) in designing IC documents may enhance IC processes. Many experts expressed the need for further research on the needs of different target groups, such as individuals with a migrant background or visual or other impairments.

**Conclusions:**

There is a significant gap between legal and ethical obligations related to IC and patients not being able to understand the abundance of unfamiliar, complex information provided to them. Evidence-based IC checklists, involving nursing staff and improved written IC materials may help improve communication in this area. Further interventional research is required to IC processes in oncology with the aim to provide optimal, patient-centred care.

**Supplementary Information:**

The online version contains supplementary material available at 10.1186/s12910-025-01348-5.

## Introduction

Obtaining informed consent (IC) is as a key ethical principle of medical research and practice highlighting healthcare providers’ respect for patients’ autonomy [1]. IC processes must cover key elements of the respective study such as aims, risks, benefits, procedures, and alternative treatment modalities [2–5]. Despite this, many challenges arise in seeking IC in routine practice. Modern medicine often requires physicians to provide patients with an abundance of unfamiliar information on rapidly evolving technologies and complex treatment options [6]. There is often a discrepancy between patients’ legal right to provide IC and their actual ability to comprehend, digest and use information related to clinical trials.

The complexity of IC is particularly challenging in oncology trials, as oncology is characterized by a remarkable diversity and speed in the development of new treatment options [7]. In recent years, the landscape of cancer treatment has undergone a comprehensive transformation, shifting from traditional approaches like chemotherapy and radiotherapy to more precise strategies such as targeted therapies, cell-based treatments, and gene therapies [8]. Modern oncology trials often involve high-stakes interventions and complex, multistage randomized designs, resulting in lengthy IC documents, and complex treatment protocols that can be overwhelming for patients [7, 9]. Furthermore, the emotional burden of a cancer diagnosis, often leading patients to experience anxiety and fear, along with clinical factors, such as metabolic disturbances, infection, and sleep dysregulation, can affect a patient’s ability to process information and make decisions [10, 11]. Oncology patients are often expected to comprehend highly complex information and make time-sensitive decisions while coping with the psychological impact of a life-threatening illness. As a result, they are confronted with an overwhelming amount of information at a moment of deep vulnerability [12]. Patients often agree to participate in a trial based on incomplete understanding of the information provided [13]. Furthermore, participants often mistakenly assume that the primary purpose of a clinical trial is their personal treatment rather than the generation of knowledge [14]. They commonly overestimate the potential benefits and underestimate the risks of trial participation compared to standard treatment. This is known as therapeutic misconception and can undermine IC, as participants do not fully grasp the true nature and purpose of the respective study [14]. Therapeutic misconception is an important challenge in obtaining IC, especially in oncology trials where patients may be vulnerable due to their health condition and hopes for effective treatment [12]. Despite efforts and initiatives to enhance IC processes [15], communicating the various components of a trial to patients without overwhelming them remains challenging [16].

Previous strategies to improve patient understanding, such as enhancing consent forms, extending IC conversations, using multimedia and teach-back methods, have shown little success [15]. Furthermore, earlier studies have shown that patients’ understanding of informed consent has not improved over the past 30 years [17]. While only very few studies have examined how IC processes could be improved in Germany [18–20] the challenges of obtaining informed consent are of international relevance, as they involve legal, ethical, communicational, and medical aspects applicable across various countries under globally recognized frameworks such as ICH-GCP and the Declaration of Helsinki [1]. Exploring the perspectives of experts from different disciplines may help to identify key challenges and potential opportunities for improving care in this area. Qualitative research methods can help to address this gap by providing novel in-depth insights into the perspectives of experts on IC processes related to cancer clinical trials. This may help to highlight critical issues and suggest strategies for enhancing how healthcare providers communicate complex IC information to patients.

## Methods

### Aim

This study explored, qualitatively, the perspectives of multidisciplinary experts on IC processes in clinical trials for cancer patients. The aim was to identify key challenges and barriers, as well as potential facilitators related to IC, in order to better understand how IC processes could be improved in oncology.

### Study design

Semi-structured phone interviews with experts from various fields were conducted. This is a sub-study of a larger project designed to develop and test strategies to improve IC processes, which also includes patient interviews and a Delphi survey.

In order to ensure a complete and transparent presentation of the research process and results, the work was based on the Consolidated Criteria for Reporting Qualitative Research (COREQ) checklist [21].

### Participants

Eligible participants were experts in the field of IC in clinical trials for cancer patients and at least 18 years of age. Experts were defined as people who were involved in obtaining IC in clinical trials, such as physicians, primarily oncologists, and study nurses; or people who had expertise relevant to IC processes, such as lawyers, members of ethic committees or researchers working in the area of IC. Patients and patient advocates involved in IC processes were also eligible to participate.

### Recruitment

Eligible participants were identified, contacted, and informed about the study by the research team using purposeful sampling. In this context, purposeful sampling referred to the intentional selection of participants based on their relevant expertise and experience related to IC processes, including a structured, nationwide search across Germany to identify potential participants. This was based on discussions among the research team which involved experts in the areas of medicine, ethics and communication science as well as targeted internet research to identify potential interviewees. Particular attention was paid to including oncologists and other experts closely linked to oncology and patient care. A total of 20 experts were invited to participate, and 17 agreed. The interviews were conducted by phone. Participants could decide on the timing of the interview according to their availabilities and individual preferences.

### Data collection

The interview guide for this study was developed based on a literature search and discussions among the research team. At the start of the interview, participants were asked to talk about their experiences with IC processes, as well as about potential challenges and strategies to improve communication in this area. for detailed information in each topic area please see the interview guide provided in Appendix 1. A member of the research team (CB) conducted the interviews between July 2021 and July 2022.

### Data analysis

The interviews were audiotaped and transcribed verbatim. One member of the research team transcribed all interviews (CB) and the transcripts were double-checked by another member of the research team. The data was processed and analysed with the help of the Qualitative Data Analysis (QDA) software ATLAS.ti [22] using framework analysis [23]. Framework analysis provides a structured method for organizing and interpreting qualitative data [23]. Initially, an inductive approach was used, meaning that one team member (CB) read through the data thoroughly and applied open coding by creating paraphrases (“codes”). These codes were then reviewed and discussed with another member of the research team (AH). Subsequently, these codes were grouped into categories by combining multiple codes related to the same topic, thereby forming an analytical framework [23]. The research group discussed the framework before it was applied to analyse subsequent interviews. In the next phase, one coder systematically coded all transcripts according to the analytical framework (CB), with oversight from another member for accuracy (AH). The framework was adjusted if new codes emerged. This iterative analysis allowed for ongoing testing and refinement of the hypotheses developed from the categories [23]. Once the categories were established and interpreted, relationships between them were explored, leading to the identification of major themes. These themes represented concepts that encapsulate and summarize essential elements within the dataset, serving as the culmination of the comprehensive analysis conducted throughout this study. All findings drawn from the data were discussed by the research team members.

## Results

Seventeen experts from 9 German cities were interviewed. Experts had a mean age of 50 years and almost half (47%) were physicians involved in clinical trials. 76% had more than 10 years of professional experience. The socio-demographic characteristics of the participants are presented in Table 1.

**Table 1:** Sociodemographic characteristics of experts

Characteristics	Experts (*n*=17)
Age (M)	50
Gender (%)	
Female	41%
Male	59%
Profession	
Physician	8
Nurse	1
Scientist	2
Patient Advocate	3
Psychologist	1
Legal expert	1
Pedagogue	1

The themes that emerged from the data were: I) the importance of effective IC conversations for informed medical decision making II) challenges resulting from role conflicts of study physicians III) two-step consent process to enhance patient understanding and decision making IV) formal requirements of IC documents jeopardizing the delivery of patient-centred care V) improved IC documents through checklists and visual aids VI) improved accessibility of trial information and greater stakeholder involvement to overcome uninformed consent.

### Empowering informed decisions: the importance of effective IC conversations for informed medical decision making

The IC conversation was perceived to be the most important part of the IC consent process. Some experts said that patients often decide to participate in a clinical trial only on the basis of the IC conversation, without reading IC documents.

There was a perception that actively involving patients in IC conversations could help them to better understand their treatment options and why their physician introduced the trial to them. They may be more likely to feel a sense of ownership over their health decisions. This may have a positive effect on study adherence, as it may foster trust and a collaborative physician-patient interaction.

*„I think that a good IC conversation also serves to build that patient-doctor or study-doctor relationship in such a way that there is a sense of ‘yes*,* I am going down this experimental path with you and the promise is that we are going down this path together and we are going to do it as a team.‘”* (Male, medical background, 37 y).

### Challenges resulting from role conflicts of study physicians

Some participants emphasised that physicians do not receive sufficient training in IC conversations. According to the experts involved in this study, IC conversations are sometimes being held like a lecture with physicians using one-way communication and overwhelming patients with complex information. Other conversations were perceived to be more like a sales pitch with physicians trying to convince patients to participate in a trial. Some experts stated that conducting an unbiased, balanced IC conversation is challenging for physicians because they often have vested interests in recruiting patients for trials. Participants may sometimes be inadequately informed about the purpose of the respective trial due to study physicians’ obligation to recruit trial participants. They may have false hopes in terms of the chance that the cancer will be cured through treatment provided as part of the trial. Further patient information, e.g. unbiased information in written format, could improve patient understanding.

*„You are not completely independent as a doctor*,* yes*,* you also have an interest in getting patients into trials*,* whether it is the funding of trials*,* I have to pay my study nurse*,* I have to be number one in recruitment*,* there are many influences on how I*,* shall I say*,* motivate a patient to go into trials. And that’s why patient information is very important as an objective tool.“ (Female*,* medical background*,* 54 y)*.

*„For me*,* this means that there is also a tension because on the one hand I have a scientific interest and want to recruit the patient for the trial*,* but I have to do this balancing act of remaining objective and somehow deciding in favour of the patient*,* even co-deciding*,* because they often don’t know. And I’m somewhere between a caregiver and a salesman.“* (Male, medical background, 37 y).

The physicians interviewed as part of this body of work said that concepts such as randomisation are often not understood by patients. According to most experts, what truly matters to patients when deciding on trial participation is the effort and potential burden required to take part in the trial. They also indicated that it would be important that patients better understand the nature of the trial and that the experimental treatment may not be successful in curing the cancer or decrease side-effects of treatment. Several experts reported that some physicians tend to simplify information, even if this compromises the completeness of the information provided. A number of experts believed that giving patients too much detailed information could increase anxiety and may thus even be harmful. They indicated that it should be communicated more clearly that trial participation primarily serves scientific purposes.

*„It’s important to really say it once: ‘You’re making a bet here*,* you might get something that could even hurt you.” (*Male, medical background, 37 y*)*.

*„When patients ask questions*,* it’s usually not ‘what do you do with the biological material?’ but ‘do I have to come in for a special appointment?’ or ‘how much blood will be taken?’ “.* (Female, medical background, 34 y)

*“If you explain too much detail about what could happen*,* you can end up talking yourself into things that then actually happen. You know what I mean?” (*Female, medical background, 43 y*)*.

### IC through a two-step consent process to enhance patient understanding and decision-making

Some experts emphasised the importance of a two-step IC process with core components of the IC being communicated by the physician in a first consultation, followed by a second visit which could be held by another member of the healthcare or research team (e.g., study nurse). This second visit could help to answer practical questions patients may have (e.g., on practical aspects related to trial participation) and sign consent forms if patients wish to participate in the trial. This would provide patients with more time to think about trial participation and read the information provided to them. It may also help them to better “digest”, recall and use this information to make informed decisions regarding their care.

*„So*,* if they are not so far along in their disease trajectory*,* they cannot really deal with this trial […]. Then you have to extend it [= the IC process] to at least two longer appointments and give them a chance to somehow deal with the fact that this is a somewhat hopeless situation.“ (*Male, medical background, 37 y*)*.

*„But what you realise is that if they (patients) get the diagnosis and the information about the trial on the same day*,* they’re completely overwhelmed*,* so they don’t get much of that information.“* (Female, medical background, 43 y).

It was also reported that many patients feel reluctant to participate in clinical trials. Study nurses may help ease this fear by providing further information on risks and potential benefits of study participation. In a trial setting, nurses are often assigned to individual patients resulting in more frequent contacts with patients than the treating physician. This may also assist with identifying and addressing patients’ needs and concerns during (and after) the trial. This may help patients digest, recall and use trial information in an emotionally challenging situation, such as after receiving information on diagnosis and treatment options, which be extremely stressful for patients. Patients commonly feel overwhelmed by receiving a myriad of complex and potentially distressing trial information, while at the same time still trying to process details provided on their health and care.

*„I think it would be a good idea to offer a follow-up IC conversation with the nursing staff afterwards so patients fully understand how everything works on our ward — when they should come*,* how long they might have to wait*,* what transportation options are available*,* how to get here*,* and how appointments are scheduled.” (*Female, medical background, 43 y*)*.

The presence of a support persons during both IC conversations was considered to be very important to help patients recall and understand the information provided and cope with anxiety and distress related to their disease and care.

*„I’m happy if the patient’s wife*,* partner*,* son or somebody else is present. On the one hand*,* it’s another form of support for the patient*,* it’s not ‘doctor against patient’*,* they have another ally. It usually makes them feel better*,* and it often gives them a chance to ask questions. Because of the emotional distance*,* support persons are often able to grasp the situation intellectually and ask further questions.“* (Male, medical background, 37 y).

### Formal requirements of IC documents jeopardizing the delivery of patient-centred care

IC documents are often too long, include too many abstract terms and the layout is commonly no designed to assist with understanding the information given.

*„And I would say that 80 to 90 per cent of the IC documents that I see as a member of an ethics committee are terrible documents. 50-*,* 60-page Times New Roman deserts with no table of contents*,* no graphics*,* no illustrations*,* no flow chart*,* no glossary*,* no structure*,* with lots of duplication. And jargon*,* with footnotes and all sorts of things that an academic can handle*,* but not the average patient.“* (Male, patient advocate, 49 y).

*„If I imagine that a patient comes to a university hospital*,* is told about a trial*,* and then wants to talk to their support persons about it*,* they’re not going to read thirty pages […].“ (*Male, patient advocate, 66 y*)*.

Sometimes many different terms are used synonymously in IC documents often leading to confusion and further contributing to lacks in understandability. This means that IC documents are often not even read by patients which was considered to be problematic because the signature on the document is a legal act forming the basis of the validity of IC processes. IC documents are heavily influenced by legal requirements to protect the rights of various stakeholders, such as the trial sponsor or the institution conducting the trial.

*„The problem is that the IC document is a very legal document. In fact*,* it is no longer a patient information at all…“ (*Male, patient advocate, 49 y*)*.

*„A 20-page IC document is useless. It is even questionable whether it is legally valid*,* because the patient is bombarded with information that they cannot grasp*,* that they cannot understand and that they are then practically forced to sign.“ (*Male, medical background, 65 y*)*.

Also, requirements related to data protection are intended to protect patients’ rights but often further increase the length and complexity of IC documents making it almost impossible for patients to understand what trial participation involves and make informed decisions regarding their care.

*„I keep criticising the seven pages on data protection in a 50-page document. But we don’t really manage to change the whole thing constructively*,* because of course if you leave out one section*,* our lawyer on the ethics committee says: ‘But it has to be in there…’.“ (*Female, educator, 37 y*)*.

*„So*,* I am legally obliged to provide that amount of information. I don’t see the legislator backing down from that.“ (*Female, lawyer, 37 y*)*.

In international multicentre trials, extensive legal regulations from the involved countries as well as the need to translate these documents often contribute to lengthy IC documents that are hard to understand and do not meet patients’ information needs. Some experts believed that some IC documents cover directive, paternalistic but often irrelevant information. This may involve extensive details on pregnancy and various forms of contraception which may not be applicable to all patient populations but make it hard for patients to understand the key messages of IC documents.

*„Of course*,* translations are a problem*,* and of course translations occur especially in multicentre trials*,* and in multicentre trials*,* from a German perspective*,* I would hope that there is somebody who is clear about what is valid in Germany and what is valid in other countries*,* because they probably often just get the IC forms from the clinical research organisations.“* (Female, scientist, 55 y).

*„A typical issue that we deal with all the time is contraception and pregnancy. On the one hand*,* it is often written in a very paternalistic way*,* i.e. it is clearly prescribed how the woman should protect herself*,* and at the same time every contraceptive method is explained in detail*,* so that the actual message is actually lost again.“ (*Male, patient advocate, 49 y*)*.

One person highlighted the difficulties faced by physicians and patients when new versions of study protocols are published, and additional consent forms are required. This is particularly problematic when studies have already been completed and patients may be asked to sign retrospectively. It is often not clear to patients what exactly has changed, which leads to confusion and increases the risk of providing “uninformed” consent.

*„What I also find really bad is that when a patient is in a trial and there are always new versions and updates*,* sometimes after trials have been completed and patients have been discharged for a long time*,* they are still expected to sign some kind of consent form retrospectively.“ (*Female, medical background, 54 y*)*.

### Improved IC documents through checklists and visual aids

Some experts emphasized that patients’ understanding of the trial is often not checked. It may be beneficial for patients and physicians to use a written evidence-based checklist during the IC conversation to help physicians cover key points and assist with checking patients’ understanding at the end of the consultation. This could also serve as a take-home resource for patients (and support persons) to remember the information discussed during the consultation and write down questions they would like to flag during their next visit.

*„I often ask: ‘Why can’t the flowchart from the trial protocol also be included in the IC document?’ There should be a page that lists all the tests and interventions vertically*,* alongside a timeline that runs horizontally. This format would allow patients to view the entire process on one page*,* clearly illustrating the chronological sequence of events and what happens at each stage.“ (*Male, patient advocate, 49 y*)*.

Many experts stated that visual aids, such as diagrams, graphs, and study flowcharts, could further improve IC documents. This may help provide an overview of important information, such as treatment options and trial procedures, making such complex information easier to comprehend. Experts also highlighted the importance of enhancing the structure and layout of the text included in IC documents. Clear headings, simple tables, and colour coding could help patients find and use information more easily. Information should also be organized more logically and prioritized to assist patients better with understanding a trial’s purpose and what to expect from study participation.

*„I would also like to see something like a standardised layout with up-to-date logos*,* I always get studies where you wonder what niche they’ve pulled this piece of paper out of*,* and for me it also has to do with respect*,* towards our patients and support persons*,* because the layout and format […] doesn’t look like something that’s been hastily cobbled together.“ (*Female, patient advocate, 54 y*)*.

The text of the IC document should be edited by further experts, such as professional translators and health communication scientists, to ensure it is understandable and meets the needs of various patient groups. Using simple, lay language was considered essential. Incorporating a first-person narrative and key questions to ask could help frame the text from the patient’s perspective, making it more personal and engaging. Additionally, short summaries and graphical abstracts could help capture attention and increase patient understanding. The importance of providing clear and understandable explanations of terms like “placebo” and other study-related terminology in a glossary was emphasized. Experts also suggested to put further effort into tailoring IC information to the needs of different target groups, such as individuals with a migrant background or those who may struggle with accessing information due to visual or other impairments.

### Improved accessibility of trial information and greater stakeholder involvement to overcome uninformed consent

From the involved experts’ points of view, it is crucial that information about trial participation is displayed publicly available on the clinics’ websites. Without this information, patients may miss research and treatment opportunities relevant to their conditions which may result in reduced recruitment success for the respective trials. Patients often rely on their clinicians to inform themselves about relevant trials. In large clinics, it can be easy for patients to overlook important trials if they are not directly informed by a physician, nurse, or other healthcare provider. Therefore, providing publicly available clear, easy-to-understand summaries of each trial would be beneficial. According to some experts, plain-language summaries could help patients to grasp the essential information about the trials listed on clinic websites, enabling them to discuss potential participation with clinic staff.

Involving patient advocates in ethics committees and engaging patient organizations early in the planning of the trial could further enhance IC documents. Patient organizations may also play a crucial role in sharing information and helping patients learn about available trials.

*„When considering trial information at an early stage*,* it is essential to involve patient organizations by stating*,* ‘We are currently planning the trial and would like to discuss the trial protocol with you. Do you see any ethical issues? Are there potential recruitment challenges? Do you think the information is being communicated effectively? What are the critical points for you?’ Engaging in this dialogue early on would be very beneficial. It prevents situations where*,* during the review process*,* patients express confusion over lengthy documents*,* saying*,* ‘I don’t understand any of this*,*’ while the trial sponsor insists*,* ‘But we need to submit it next week’.“ (*Male, patient advocate, 49 y*)*.

Early involvement of local ethics committees in the conceptualizing and planning trials was essential for many experts, as it may foster greater engagement in and understanding of the relevant trials. However, it has been noted that many ethics committees lack patient advocates, and those who are present are often inadequately trained to judge IC documents. Increasing public awareness and providing training for patient advocates could further enhance their understanding of their roles and help them contribute to patient-centred IC information. Additionally, it is important to involve patient advocates from various socio-demographic backgrounds, including those with medical knowledge and those without. Many experts suggested that ethics committees take a more active role in improving IC information by not merely approving trial protocols and IC documents but setting stricter requirements for clarity and understandability of IC information.

*„This is a purely political question*,* we have to activate the competent authorities*,* that is BfArM (Federal Institute for Drugs and Medical Devices) and the ethics committees*,* so that they no longer simply wave these IC documents through. They all get waved through and the companies of course say: If BfArM and the ethics committee accept it*,* why should we do it any other way?.“ (*Male, medical background, 62 y*)*.

## Discussion

IC conversations are a crucial component of the overall IC processes, especially since many patients do not read IC documents. These conversations may feel overwhelming or persuasive to patients, given that they involve an abundance of complex information which may not be provided in an unbiased way since physicians have vested interests in recruiting patients to trials. Providing trial information in an understandable and balanced way is essential to support patients with making informed healthcare decisions. Experts involved in this study also highlighted the importance of a two-step IC process, where physicians provide information about trial participation in a first consultation, followed by a second session with another member of the healthcare team, e.g. a study nurse. This approach is especially relevant in oncology trials, where patients face high emotional and cognitive load when making participation decisions. This could also help to address questions patients may have, discuss practical aspects, such as additional clinic visits, and improve patients’ overall understanding of trial information. It would also give patients more time to process trial information and discuss them with their support persons if they wish this. Furthermore, introducing IC checklists may help clinicians to ensure that all key aspects of a trial are covered and verify whether patients understood them. Graphical abstracts, along with short videos and microlearning tools, could further support information delivery through brief and accessible visual summaries of key aspects of the respective trial. While our study focused on oncology, the identified communication challenges and proposed strategies may be relevant to other medical fields where patients must process complex trial information under emotionally challenging circumstances. Further research is needed to address the specific needs of diverse target groups, including migrant populations and those facing challenges in accessing information due to visual or other impairments to enhance IC processes.

### Challenges of IC documents

Over the last three decades, the average length of IC documents has increased tenfold further decreasing patients’ ability to understand key aspects of the respective trial [7]. While many patient advocates argue for increased patient autonomy and understanding, legal obligations foster adherence to formal criteria [24]. Although legislations such as the German Medicinal Products Act (§ 40b AMG), the German Civil Code (§ 630e BGB), the Clinical trials - Regulation EU No 536/2014 and the Declaration of Helsinki aim to ensure comprehensive patient information and protect patients’ rights, IC documents have become highly legalistic, potentially prioritizing the protection of sponsors and institutions over patient understanding [25]. They require large amounts of complex information to be delivered to patients jeopardizing the understandability of IC documents [26]. As a result, patients commonly sign these documents without understanding the core components of the respective trial, which undermines their right to make informed decisions regarding their care and may induce additional anxiety and frustration [17]. This tension between legal formalism and patient understanding raises important ethical concerns. The Declaration of Helsinki places a strong emphasis on providing participants with sufficient, comprehensible information, enabling them to make a voluntary and informed decision regarding their participation in a trial [1]. Our findings suggest that the current practice of overly complex IC documents falls short of this standard, thereby undermining the ethical principle of IC. Furthermore, the Declaration encourages meaningful engagement of patients and the public throughout the research process [1]. The active involvement of various stakeholders, including patient organizations, in the planning and design of clinical trials could help to identify ethical concerns at an early stage of the planning process [27]. This could improve the understandability of study materials and increase patients’ trust in medical research.

### Enhancing IC processes

A two-step IC process, involving an initial IC conversation and a subsequent visit one to two weeks later could allow patients more time to understand, recall and use trial information [12, 28]. This approach is particularly valuable in oncology trials, where patients commonly face high levels of emotional stress and many treatment decisions are probabilistic and preference-sensitive [29]. A two-step IC process could help them to make informed healthcare decisions [28]. However, this approach is not routinely implemented in clinical practice [30], and time constraints may limit its feasibility in certain contexts, for example when treatment must begin promptly. In such cases, the interval between both consultations may need to be shortened. Nevertheless, dividing the conversations may contribute to greater structure and conciseness. Indeed, interventional trials measuring consultation time have demonstrated that structured communication interventions do not necessarily prolong consultations and may even improve efficiency [31]. To facilitate the use of a two-step IC process, the second consultation could be conducted by nursing staff face-to-face or online to help patients to clarify further questions and concerns. This may also help overcome patients’ fears related to clinical trials [32, 33], e.g. by nurses emphasizing the more intensive care provided within a trial due to increased clinic visits and improved care coordination [34]. Providing patients with sufficient time, opportunities for reflection, and verification of understanding is essential to respect their autonomy and to ensure that consent is voluntary and informed, as demanded by international ethical guidance such as the Declaration of Helsinki [1].

Checklists for IC conversations could serve as a communication guide and help physicians to verify that all key aspects of the respective trial, such as aims, risks and benefits, right to withdraw, randomization and alternative treatment were addressed during the IC conversation. The checklist could serve as a tool for quality control which could be adapted to the respective study context. It could also be handed to patients to support them with following the IC conversation and serve as a take-home resource to increase information recall [35]. An increased patient understanding of study methodology and the risks and benefits associated to the respective trial may also increase their willingness to participate [36]. The checklists for IC conversations could be developed collaboratively by healthcare professionals, patient advocates, regulatory bodies, and interdisciplinary research teams to ensure they are easy-to-use, comprehensive and tailored to the needs of both patients and healthcare providers. Once developed, these checklists could be tested with the help of interventional research to evaluate their feasibility and effectiveness.

### Enhancing IC documents

IC documents are often lengthy, complex and poorly structured, making them difficult to understand and process [17]. The use of medical jargon, inconsistent terminology and a lack of visual aids further impacts on the understandability of these documents [37, 38]. Brief and clearly marked headings, simple tables and color-coding may help patients to locate and understand key aspects of the respective trial. For example, a graphical abstract could be used to visually present the core elements of a trial using an image to summarise aims, risks and benefits of the respective study, making this information more accessible to heterogeneous target groups, including non-academic audiences [39]. Guidelines for developing graphical abstracts have been developed [39] and could be adapted to IC processes for clinical trials. This may be more effective in improving patient understanding than plain language summaries [40]. Also microlearning tools, dividing information delivery into smaller episodes and skill elements, could help to increase the accessibility of the IC document [41]. Micro learning involves minimal time consumption and operating expense, and can be part of a modular learning setting that patients can access according to their needs and thus at times of their choosing [42].

Also, IC documents often include comprehensive details on topics not applicable to all patient groups (e.g. contraceptive methods). This increases the length of IC documents. It may further hinder patient understanding and their ability to make informed healthcare decisions, given that there is evidence to suggest that patients often only remember a fraction of the information provided in IC documents [17]. When being overwhelmed with overly detailed and/or irrelevant content, patients may struggle to determine what is most important, highlighting the need to tailoring IC information to the needs of different patient groups. Artificial intelligence (AI), along with technologies like machine learning, natural language processing, predictive analytics, chatbots, and telemedicine could be used to extract relevant information from patients’ medical records and tailor IC information to their specific situation and needs. Such technology is already being used to improve recruitment in oncology clinical trials, e.g. by increasing efficiency, cost savings, improving recruitment, accuracy, patient satisfaction, and creating user-friendly interfaces [43]. Tailored IC documents may also increase trial participation by individuals with diverse sociodemographic backgrounds, such as those with a migration background or cognitive impairments. Further research is required to explore the information needs of various target groups and how they could be addressed to further enhance the accessibility of clinical trials.

### Role conflicts of study physicians

The central role of IC conversation as part of the overall IC processes was emphasized in our study. Many patients make decisions on study participation based on IC conversations rather than reading through IC documents [35, 44]. IC conversations are also important for building trust and partnership between physicians and patients, which can increase patient adherence during the trial and decrease drop-out [35]. During IC conversations, physicians are supposed to inform about the risks and benefits of a study in an unbiased manner, while also having an interest in increasing patient recruitment [45]. Physicians need to navigate these potential conflicts of interest and clearly communicate the experimental nature of clinical trials and the possibility of no benefit or even harm. For example, when a physician who is treating a patient is also seeking consent for a trial he or she is conducting, the patient may feel pressured to participate in order to maintain or foster their physician-patient relationship [46, 47]. Efforts should be made to avoid this, e.g. by having someone other than the treating physician obtain IC [46]. In oncology, where treatment decisions are often urgent and emotionally charged, minimizing undue influence is especially critical. The Declaration of Helsinki [1] explicitly warns against such undue influence, emphasizing that participation in research must always be based on free choice.

### Strengths and limitations

To the best of our knowledge, this is the first study to explore the perspectives of multidisciplinary experts on IC processes in clinical trials for cancer patients. We used an interdisciplinary approach integrating medical, ethical, legal, and communicational perspectives. Our findings help to develop evidence-based strategies to improve IC that could be readily implemented into routine care. They may also be transferable to areas of medicine other than oncology, bringing momentum to a research field where progress is urgently needed.

We recruited experts from various disciplines and with heterogeneous sociodemographic backgrounds. This was an explorative, hypothesis-generating study. Thus, the generalisability of the study findings is limited due to the sample size. Furthermore, certain professional groups (e.g., nurses) were underrepresented compared to physicians. Future studies should involve more legal experts and representatives from the pharmaceutical/biotech industry. Also, the definition of who qualifies as an expert for this study partly relied on individuals’ self-assessment.

## Conclusion

IC processes are often overwhelming for patients which hinders their ability to make informed decision regarding their care. Current legislation such as the as the German Medicinal Products Act (§ 40b AMG), the German Civil Code (§ 630e BGB), the Clinical trials - Regulation EU No 536/2014 and the Declaration of Helsinki aim to protect patients’ rights but commonly increase the length and complexity of IC documents jeopardizing patient understanding of the trial they could join. Physicians may experience conflicts of interest when having to present trial information in an unbiased manner while also being obliged to increase patient recruitment to the respective trial. A two-step IC process could help to improve patient understanding of trial information and increase their willingness to participate in medical research. Incorporating checklists and visual aids, such as graphical abstracts, could further enhance the understandability and accessibility of IC information. Additionally, AI could be used to tailor IC information to individual patient needs. Future interventional research should test the effectiveness and implementability of these strategies.

## Supplementary Information

Supplementary Material 1.

Supplementary Material 2.

## Data Availability

Qualitative data are available from the corresponding author upon reasonable request.
