# Supplementary material for: How to improve informed consent processes in clinical trials with cancer patients: a qualitative analysis of multidisciplinary experts’ perspectives
Source: BMC Med Ethics. 2025 Dec 2;26:171. doi: 10.1186/s12910-025-01348-5 (PMC12690889; doi:10.1186/s12910-025-01348-5)
Supplement: Supplementary file 1 — Supplementary Material 1. [file 12910_2025_1348_MOESM1_ESM.docx]

As already mentioned, my name is xy and I work as a scientist at the University of Regensburg.
We are currently conducting a research project on informed consent in cancer clinical trials.

Therefore, I would like to ask you to tell me what problems you see with informed consent for clinical trials in oncology.

You can feel completely free to say anything that comes to mind and talk for as long as you like. I will listen to you carefully and make notes of any questions that we may need to clarify later.

We can stop the interview anytime or skip questions you don’t feel comfortable answering. Please just let me know when you feel you would like to stop or skip questions.

Now, I would like you to tell me about your perspective on the informed consent process, in as much detail as you like, and you can take as much time as you like.

Understanding of information

- In your experience, do patients fully understand the information provided to them during the informed consent conversation?
  - What factors help or hinder their understanding?
  - Have you noticed differences between patient groups?
- Which parts of the information do you think patients tend to understand the least?
  - Are there specific concepts, terms, or sections that cause confusion?
  - Do you think complexity or the volume of information plays a bigger role?
- Informed consent documents often contain a large amount of information, and studies suggest that patients may not fully understand it or may forget parts of it shortly after the conversation. Do you consider this to be an ethical/legal problem?
  - How do you think this impacts patient autonomy?
  - Should the process be adapted to address this issue?

Specific challenges in study-related informed consent

- Where do you see particular challenges when obtaining informed consent for clinical studies?
  - Involvement of third countries
  - Data protection and privacy
  - Patients’ attention span or fatigue
  - Language or cultural barriers

Strategies to improve the informed consent

- What measure could help to overcome these challenges?
  - Tools or formats that aid understanding
  - Timing and setting of the consent discussion
  - Training for study staff
- How do you think information should ideally be presented to patients? Is this feasible in practice?
- Are there any other strategies that could help during informed consent discussions with patients?
- Which of these informed consent strategies would you consider?
  - Video informed consent in the clinic, plus conversation with doctor
  - Video informed consent at home online, after conversation with doctor
    - Brochure with flowcharts, graphs and tables, to be read at home after the informed consent conversation
    - Electronic informed consent
    - Informed consent summary (1200-1800 words) with key information and the full consent form as an appendix
    - Extended informed consent conversation (with a member of the study team or with a neutral person - peer to peer)
    - For patients with a migration background: educational materials available in additional languages.

Do you have any other comments you would like to make? Do you have any questions?

Thank you!
