# Supplementary material for: How to improve informed consent processes in clinical trials with cancer patients: a qualitative analysis of multidisciplinary experts’ perspectives
Source: BMC Med Ethics. 2025 Dec 2;26:171. doi: 10.1186/s12910-025-01348-5 (PMC12690889; doi:10.1186/s12910-025-01348-5)
Supplement: Supplementary file 2 — Supplementary Material 2. [file 12910_2025_1348_MOESM2_ESM.pdf]

Consolidated criteria for reporting qualitative studies (COREQ): 32-item checklist

No. Item	Guide questions/description	Reported on Page #
**Domain 1: Research team and reflexivity**		
Personal Characteristics		
1. Interviewer/facilitator	CB	4
2. Credentials	At the time of the interviews, CB held an M. Sc. degree und was a PhD Student.	/
3. Occupation	She was working as a Research Associate at the University of Regensburg when the interviews were conducted. The other researchers held clinician and/or scientist faculty positions.	/
4. Gender	The interviewer was female.	/
5. Experience and training	CB has extensive experience in the healthcare field, having worked as a nurse for six years. Furthermore, she has been involved in several other qualitative research projects.	/
Relationship with participants		
6. Relationship established	The interviewer had no prior relationship with the participants.	4
7. Participant knowledge of the interviewer	The interviewer introduced herself to participants, stating her research fellow status and affiliation. The purpose of the study was discussed with the participants.	4
8. Interviewer characteristics	The interviewer was a health services researcher with nursing background.	/

**Domain 2: study design**		
Theoretical framework		
9. Methodological orientation and Theory	The methodological orientation was qualitative content analysis, applying the framework analysis approach as described by Gale et al. (2013)	4-5
Participant selection		
10. Sampling	We used purposeful sampling.	4
11. Method of approach	E-Mail	4
12. Sample size	17	4
13. Non-participation	3, due to time constraints	4
Setting		
14. Setting of data collection	The interviews were conducted by phone.	4
15. Presence of non-participants	Only the participant and interviewer were present during the interview.	/
16. Description of sample	Participants were experts in the field of IC in clinical trials for cancer patients and at least 18 years of age.	4-5
Data collection		
17. Interview guide	Semi-structured, tested with the study team through two pilot interviews.	4
18. Repeat interviews	No	/
19. Audio/visual recording	Audio recording	4
20. Field notes	No	/
21. Duration	30-45 Minutes	/
22. Data saturation	Yes	4
23. Transcripts returned	No	/
**Domain 3: analysis and findings**		
Data analysis		
24. Number of data coders	2	4-5
25. Description of the coding tree	Initially, an inductive approach was used, meaning that one team member (CB) read through the data thoroughly and applied open coding by creating paraphrases	4-5

	(“codes”). These codes were then reviewed and discussed with another member of the research team (AH). Subsequently, these codes were grouped into categories by combining multiple codes related to the same topic, thereby forming an analytical framework.	
26. Derivation of themes	Inductive and deductive approach	4-5
27. Software	ATLAS.ti	4
28. Participant checking	No	/
Reporting		
29. Quotations presented	Yes, quotations are presented in a de-identified fashion.	5-10
30. Data and findings consistent	Consistency between the data and the findings exists.	5-10
31. Clarity of major themes	Major themes are clearly identified and described.	5-10
32. Clarity of minor themes	Minor themes are clearly identified and described.	5-10
